# The Child Behaviour Assessment Instrument: development and validation of a measure to screen for externalising child behavioural problems in community setting

**DOI:** 10.1186/1752-4458-4-13

**Published:** 2010-06-08

**Authors:** Diana C Samarakkody, Dulitha N Fernando, Hemamali Perera, Roderick J McClure, Hiranthi De Silva

**Affiliations:** 1 Department of Community Medicine, Faculty of Medicine, University of Colombo, Kynsey Road, Colombo 8, Sri Lanka; 2 Monash University Accident Research Centre, Monash University, Clayton, Victoria 3800, Australia; 3 Department of Psychological Medicine, Faculty of Medicine, University of Colombo, Kynsey Road, Colombo 8, Sri Lanka; 4 Mental Health Unit, Ministry of Healthcare and Nutrition, 385, Colombo 10. Sri Lanka

## Abstract

**Background:**

In Sri Lanka, behavioural problems have grown to epidemic proportions accounting second highest category of mental health problems among children. Early identification of behavioural problems in children is an important pre-requisite of the implementation of interventions to prevent long term psychiatric outcomes. The objectives of the study were to develop and validate a screening instrument for use in the community setting to identify behavioural problems in children aged 4-6 years.

**Methods:**

An initial 54 item questionnaire was developed following an extensive review of the literature. A three round Delphi process involving a panel of experts from six relevant fields was then undertaken to refine the nature and number of items and created the 15 item community screening instrument, Child Behaviour Assessment Instrument (CBAI). Validation study was conducted in the Medical Officer of Health area Kaduwela, Sri Lanka and a community sample of 332 children aged 4-6 years were recruited by two stage randomization process. The behaviour status of the participants was assessed by an interviewer using the CBAI and a clinical psychologist following clinical assessment concurrently. Criterion validity was appraised by assessing the sensitivity, specificity and predictive values at the optimum screen cut off value. Construct validity of the instrument was quantified by testing whether the data of validation study fits to a hypothetical model. Face and content validity of the CBAI were qualitatively assessed by a panel of experts. The reliability of the instrument was assessed by internal consistency analysis and test-retest methods in a 15% subset of the community sample.

**Results:**

Using the Receiver Operating Characteristic analysis the CBAI score of >16 was identified as the cut off point that optimally differentiated children having behavioural problems, with a sensitivity of 0.88 (95% CI = 0.80-0.96) and specificity of 0.81 (95% CI = 0.75-0.87). The Cronbach's alpha exceeded Nunnaly's criterion of 0.7 for items related to inattention, aggression and impaired social interaction.

**Conclusions:**

Preliminary data obtained from the study indicate that the Child Behaviour Assessment Instrument is a valid and reliable screening instrument for early identification of young children at risk of behavioural problems in the community setting.

## Background

Child psychiatric problems are recognized as emerging public health issue throughout the world suggesting a global prevalence of approximately 20% [1]. Behavioural problems are the commonest psychiatric problem among young children [1]. In Sri Lanka, prevalence of behavioural problems among children is reported as 27.2% at the clinical setting accounting second highest category of the psychiatric problems [2]. Young children with behavioural problems are at a greater risk of developing psychiatric disorders in later life [3,4] and contribute disproportionately to the substantial social and economic burden attributable to mental health problems in the community [5].

The major mediating factors on the pathway to behavioural problems interact with each other over time, and are amenable to effective intervention before problems are stabilized [3]. Because these disorders have a good prognosis if treated at their onset, early identification of such disorders and referral for appropriate care provides an excellent opportunity to improve the mental health of populations [3]. However, early identification and differentiation of such behaviours from normal behaviours is challenging due to the complex and slow rate at which these behaviours manifest and the high overlap of diagnostic categories [6-8]. More over many of the problem behaviours evident during this period are, to some extent, normative and simply reflect developmental changes and stressors [3,9]. Thus the parents and health care workers have limited ability to identify behavioural problems at an early stage and most children are referred only at an advanced stage of the problem behaviour spectrum [6-8].

Previous studies have shown that childhood behavioural problems are generally first evident in 4-6 years age group [3,4] during which many children may not present at the clinical setting [8]. Thus screening for behavioural problems targeting this age group at the community level would enhance the early recognition and referral for appropriate care. Furthermore as assessment at the community level can be performed without the direct involvement of a health professional, screening at the community level can be considered more inexpensive than screening at the clinical screening.

Although several instruments exist to identify behavioural problems of children [10-16] most instruments are lengthy, complex, time consuming and have a requirement to be personally administered by trained staff with adherence to specific instructions [17,18]. The disadvantages inherent to these instruments make them inappropriate for use as routine community screening tools. Thus, there is urgent need for the development of a simple behaviour screening instrument with good psychometric properties that can be used at the grass root level.

The objectives of this study were to develop a screening instrument for early identification of behavioural problems among children aged 4-6 years in the community and to validate the instrument for use in the community setting.

## Methods

This study was conducted in 2 phases: 

**Phase 1**. Development of the screening instrument by: (a) defining the behavioural problems intended to screen (b) reviewing the available literature for possible items of child behaviour screening instrument (c) constructing a preliminary list of items based on literature review (d) ascertaining the final instrument using Delphi technique.

**Phase 2. **Assessment of validity and reliability of the final instrument

### Phase 1: Development of the Instrument

#### (a) Definition of the behavioural problems intended to screen by the instrument

Behavioural problems of children was defined by considering the different definitions given by other authors [19-24], and by conducting review discussions with several experts in the fields of Pediatrics, Child Psychiatry, Community Medicine and Child Psychology. This definition was based on identification of behavioural problems of children aged 4-6 years that fulfil the following criteria.

1. Behaviours which give rise to significant disturbance to the psychological well being and the future life of the child.

2. Behaviours that need early intervention by professionals and the early intervention result in good prognosis.

Based on the above criteria, behavioural problems of children aged 4-6 years was defined in this study as: behaviours which seriously limit or delay access to and use of ordinary society and carry significant disturbance for child's current and future psychiatric status [19].

According to the definition the intended screening instrument would contain six domains: inattention, hyperactivity and impulsivity, aggression, impaired social interactions, abnormalities of communication and restricted, stereotyped pattern of behaviour [19-24]. It is acknowledged that most of the behavioural problems that resolve with time, without any special intervention will not be detected by this instrument.

#### (b) Reviewing the available literature for possible items of child behaviour screening instrument

A a systematic search for items used in other available study instruments and published literature was undertaken on the databases listed in Medline and PsycLit and other sources such as text books on psychiatry [19-24] information sheets, scoring forms, manuals and personal communication with the authors/publishers. Screening instruments for child behaviour problems and early symptoms of child behavioural problems were the key terms used in this search.

#### (c) Constructing a preliminary list of items based on literature review

Following the literature review authors constructed a preliminary list of items covering six domains of the definition of problem behaviour. This contained 54 items each describing a potential action that a child of 4-6 year age with problem behaviour would perform. The items conform to the definition of problem behaviour were included without prior judgement on their relevance by the authors.

#### (d) Ascertaining the final instrument using Delphi technique

The final instrument was ascertained from the preliminary list of 54 items using the Delphi technique. A panel of 15 experts in the areas of Community Medicine, Child Psychiatry, Paediatrics, Child Psychology and policy making were recruited. They were informed the objectives of the study and the definition of the behavioural problems that are intended to be identified using the instrument. They were told that the instrument was being developed to be administered by a lay interviewer or primary health care worker to the mother or the care giver of the child as a routine screening activity, in the community. The need for a simple and concise instrument was highlighted. To gain consensus of the above experts, three rounds of rating were carried out. During the first round, an open ended questionnaire was prepared on the preliminary list of 54 items. Then the participants were asked to rate each item on a five point scale as: 1. Most important; 2. Important; 3. Don't know; 4. Unimportant; 5. Should be deleted, with regard for inclusion in the screening instrument and give any comments or generate more items based on the objectives and definition used in the study. Items rated as "Most important" or "important" by more than 75% of the panel members and accepted new items generated by them were selected for the second round of rating. At the end of this round items positively rated by 80% of panel members were selected and suggested modifications were done accordingly [25]. The final instrument which consisted of 15 items measuring six domains namely inattention (items 1,2 & 5), hyperactivity and impulsivity (items 3,4 & 6), aggression (items 7,8 & 9), impaired social interactions (items 10,11&15) abnormalities of communication (item 14) and restricted, stereotyped pattern of behaviour(items 12&13), was developed following the third round, and named as the Child Behaviour Assessment Instrument (CBAI). The average time taken for administration of the CBAI was five minutes.

An open ended question regarding the presence of any other behavioural problems was included at the end of the questionnaire to obtain other significant behaviours not included in the instrument. The response choices for each of the 15 items were provided as; "very often", "some times" and "never" and the scoring of 2, 1, 0 attributed to each of the these categories such that the lower value (0) indicated a lesser likelihood of having a behavioural problem where as the higher value (2) indicated a higher likelihood of having a behavioural problem. To ensure internal consistency of the questionnaire some of the items (1, 5, 10, 11, 14, and 15) were worded to assess positive behaviours and the responses were reversed scored. To quantify overall impact of each component of the above instrument, simple, unweighted count of event score, was developed by adding the individual score for each item which was ranging from 0 to 30 [26].

See the Additional file 1 for the CBAI including instructions for interviewers.

Translation of the developed instrument into local (the Sinhala) language was undertaken by a panel of individuals who were fluent in both English and Sinhala [27]. They translated the instrument independently of one another, using clear simple language to cater the respondents. Then each item of the original English version of the instrument and its corresponding five translations were considered at a time for evaluation and consensus generation following which final translation achieved.

Pre testing of the developed instrument was carried out on a convenience sample of 50 mothers of children aged 4-6 years. Clarity and relevance of the items were assessed and certain modifications were made accordingly.

### Phase 2: Assessment of validity and reliability of the final instrument

#### Study design, sample population and study setting

Validity and reliability of the final instrument was assessed by conducting a validation study using repeated measures within subject design. Using a two tier randomization process a representative community sample of 332 male and female children between the ages of 4-6 years was recruited from the Medical Officer of Health (MOH) area Kaduwela, Sri Lanka. Kaduwela is a semi urban MOH area in the District of Colombo, Western Province, Sri Lanka with a population of 209,502 with a diverse range socio demographic and ethnic composition. Children whose parents or care givers can comprehend Sinhala were included in the sample as the Sinhala translation of the developed instrument was used for data collection. Children with diagnosed behavioural problem or chronic neurological diseases (epilepsy and cerebral palsy) that may influence the behavioural pattern, confirmed by a diagnosis card, children living in institutions (Hospitals, Orphanages) and children who were acutely ill at the time of interview were excluded from the study.

## Procedures

### Validity assessment

For assessment of validity of the instrument participants underwent two procedures of data collection on (i) assessment of behaviour status by an interviewer, administering the CBAI on the mother or the principal care giver of the eligible child (ii) assessment of behaviour status by a clinical psychologist following clinical interviews with both the mother and the child based on DSM IV criteria [23].

The measures were obtained on the same day and the order of behaviour assessment by the interviewer or the clinical psychologist of a particular child was randomly selected to minimize response bias. The two assessments were done blindly and independently. At the end of each assessment the total CBAI score for the child was obtained and the clinical psychologist recorded the behaviour status (whether the child is having a behaviour problem or not) of the child on a clinical record sheet. Behavioural diagnosis of the clinical psychologist was considered as the gold standard.

Face and content validity were qualitatively assessed by experts in the fields of Community Medicine, Psychological Medicine and Paediatrics by appraising the extent to which the conceptual definition has been appropriately translated into operational terms [28].

### Reliability assessment

To assess the reliability of behavioural assessment of children using the CBAI, repeated measurements were obtained by administering the CBAI on a randomly selected sub sample of 50 (15%) respondents. In order to minimise recall error data were obtained following two weeks interval from the initial assessment.

### Statistical analysis

Sensitivity and specificity for each possible CBAI score were calculated considering the assessment of the clinical psychologist as the gold standard [29]. To determine the optimal screen cut off point of the instrument, Receiver Operative Characteristic (ROC) curve analysis was performed by plotting sensitivity against 1- Specificity in relation to the different cut off points [29].

Construct validity was assessed by testing whether the CBAI scores were consistent with what would be expected if the developed instrument was a valid measure of problematic child behaviour. A null hypothesis was proposed that stipulated the mean score of the children with behavioural problems should not differ significantly from that of children without behavioural problems (normal behaviour). The mean score of the children identified as having problem behaviour by the Clinical Psychologist was compared to that of the children identified as having normal behaviour, using one way ANOVA.

Reliability of the instrument was appraised by two techniques: Test -retest reliability and internal consistency analysis [28,29]. The exact agreement between the scores obtained two weeks apart on the subsample was compared by intra class correlation coefficient analysis [28,30].Internal consistency was appraised by using Cronbach's alpha coefficient, which is the measure of the overall correlation between items within an attribute [28,29]. All statistical analysis was performed with SPSS statistical software (SPSS Inc, USA).

### Ethics

Ethical clearance was obtained from the Ethical Review Committee, of the Faculty of Medicine, University of Colombo, Sri Lanka and informed consent was obtained from parents or the legal guardians of all participating children.

## Results

### Sample

A sample of 332 children aged 4-6 years were recruited for the validation study. There were 61(18%) children in this sample classified as having problem behaviour with significantly higher proportion of males (Table 1).

**Table 1:** Characteristics of the sample (N = 332)

Variable	Problem behaviour N (%)	Normal behaviour N (%)	Chi-square p value
**Gender**			
Female	22 (36.1)	154 (56.8)	.003
Male	39 (63.9)	117 (43.2)	
**Ethnicity**			
Sinhalese	40 (65.6)	171 (63.1)	.725
Tamil	10 (16.41)	39 (14.4)	
Other	11 (18.0)	61 (22.51)	
**Child going to preschool/school**			
Never gone to preschool/school	16 (26.2)	60 (22.1)	.788
Going to kinder/preschool	20 (32.8)	95 (35.1)	
Going to primary school	25 (41.0)	116 (42.8)	
Monthly family income (LKR*)			
Less than.5000	24 (39.3)	5 (1.8)	.000
5001-10,000	11 (18.0)	36 (13.3)	
10,0001-20000	13 (21.3)	145 (53.5)	
20,001-40,000	6 (9.8)	48 (17.7)	
More than 40,000	7 (11.5)	37 (13.7)	

* Sri Lanka Rupees

### Validity of the screening instrument

**Figure 1:**
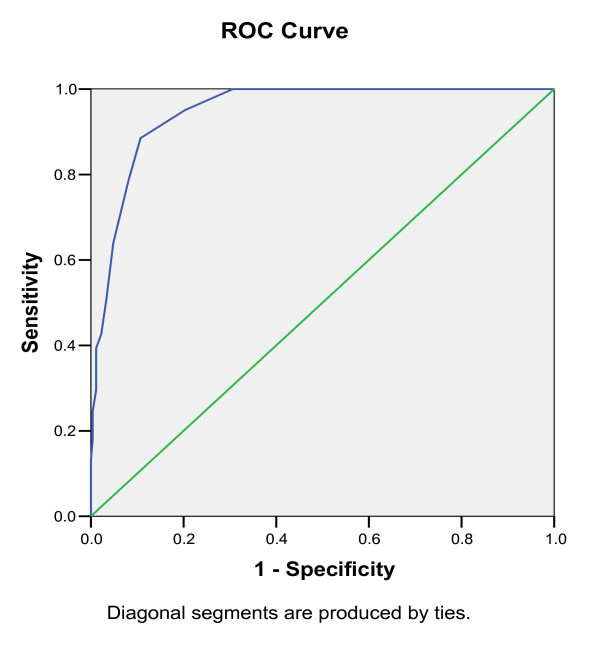
**ROC curve of CBAI score in the sample**.

Table 2 shows the sensitivity and specificity at selected possible CBAI score. The ROC curve of CBAI total score in the sample is presented in Figure 1. Considering Table 2 and Figure 1, the optimal score of discrimination between problem behaviour and normal behaviour children was identified as > 16. This score provided a sensitivity of 0.88 (95% CI = 0.80-0.96) and specificity of 0.81 (95% CI = 0.75-0.87).

**Table 2:** Sensitivity and Specificity at selected CBAI Score in the sample

CBAI Row Score	Sensitivity	Specificity
1	1.000	.004
4	1.000	.056
8	1.000	.191
12	1.000	.479
13	1.000	.517
14	1.000	.674
**15**	**.951**	**.787**
**16**	**.885**	**.815**
17	.787	.913
18	.705	.932
19	.639	.948
20	.508	.963
24	.246	.993
28	.049	1.000
29	.016	1.000
30	.000	1.000

For this sample the positive predictive value at this cut off point was 51.92 and the negative predictive value was 96.92 (Table 3). The area under the ROC curve was calculated as 0.949 (95% confidence interval is 0.926-0.972). This shows that the instrument can satisfactorily discriminate the children having behavioural problems from those with normal behaviour.

**Table 3:** Cross tabulation of CBAI score and assessment of clinical psychologist (gold standard) at the cut off score of > 16

	Assessment by clinical psychologist (Gold standard)		
		
Assessment based on the questionnaire	Problem behaviour N (%)	Normal behaviour N (%)	Total N (%)
Problem behaviour	54 (88.20)	50 (18.46)	104 (31.33)
Normal behaviour	7 (11.47)	221(81.54)	228 (68.67)
Total	61 (100.00)	271 (100.00)	332 (100.00)

Sensitivity = 54/61 × 100 = 88.52

Specificity = 221/271 × 100 = 81.54

Predictive value positive = 54/104 × 100 = 51.92

Predictive value negative 221/228 × 100 = 96.92

The results of one way Analysis of Variance demonstrated that the children with behavioural problems had a significantly higher mean score (21.377) compared to that of children without problem behaviour (7.040) (p = 0.001).

### Reliability of the screening instrument

Reliability analysis showed a satisfactory agreement between the test and retest scores

(Intra Class Correlation Coefficient of 0.851, with 95% CI of 0.731-0.971). Cronbach's alpha exceeded Nunnaly's criterion of 0.7 for items measuring inattention, aggression and impaired social interaction implying satisfactory correlation [31]. (Table 4)

**Table 4:** Internal consistency of CBAI

Construct of CBAI	Cronbach's alpha
Inattention	.761
Hyperactivity and impulsivity	.651
Aggression	.765
Impaired social interaction	.710
Abnormalities of communication	.691
Restricted, stereotyped behaviour	.658

## Discussion

The main contribution of this study was the successful development of a valid and reliable screening instrument to assess the behavioural problems of children in the community setting. Based on the CBAI score at a cutoff point of 16 or above, 18% of the community sample of children were identified as having a problem behavior. Previous studies have shown satisfactory reliability of maternal and caregiver ratings of behavioural problems of preschool children [32,33] and the observed prevalence of problem behavior in this sample was consistent with previously reported results [34,35].

We used several complementary methods to assess the validity of the CBAI, as this provides the most accurate assessment of psychometric properties [28]. Assessment of the face and content validity of the instrument confirmed the conceptual definition had been appropriately translated into operational terms.

The CBAI's sensitivity of 0.88 and specificity 0.81 at the cut off point of >16 compares well with the criterion validity of the other behavioural screening instruments in primary care settings [10-15]. Strength of this study was that the validity of the CBAI was demonstrated in a sample drawn from the community. The fact that the study sample was similar to the population in which the screening instrument was intended to be used resulted in an uninfluenced validation of the instrument, which was not over estimated by extremes of cases nor under estimated by volunteers [29]. Results of the construct validity assessment showed a highly significant difference (p < 0.001) between the CBAI scores obtained by two groups.

These results showed that the Child Behaviour Assessment Instrument (CBAI) is a valid screening instrument. More over as this screening instrument is simple, non invasive, easy to administer imposing minimal discomfort on the children and care givers, can be considered as a test that could be recommended for use at the community level.

## Limitations

This study has several methodological limitations that need to be considered in the interpretation of the results. Although this instrument is intended to screen externalising problems of children aged 4-6 years, we acknowledge that there may be a possibility of leaving out children with internalising problems. However as the internalising problems are considered primarily disorders of adults with some times have late childhood onset, the proportion of undetected children with internalising problems can be considered as minimal [20-23].

A major limitation of this instrument is its retrospective nature of the mothers and caregivers responses on behaviour of the children, introducing the possibility of recall error. However it is likely that this error will be non-differential with respect to the blinded assessment of the Clinical Psychologist and therefore would not have affected the validation assessments undertaken in the study. Previous research suggest that although there is likely to be under reporting due to recall error, responses generally accurately reflect the occurrence of adverse childhood behaviour[36].

The approach employed to assess the reliability of the Child Behaviour Assessment Instrument was to re-administer the instrument to a randomly selected sub sample of 50 (15%) respondents. The major draw back in the test-retest method is the possibility that, during the interval between the two tests occasions the behaviour pattern may change resulting in a lack of consistency that reflect a true change in the subject, rather than a lack of instrument precision. More over, the respondent may remember the responses of the first occasion and simply repeat them on the second occasion, inflating the estimates of the consistency of the responses. In the present study, the CBAI was re-administered after two weeks to balance these potential effects of errors.

Cronbach's alpha as a measure of internal consistency exceeded Nunnaly's criterion of 0.7 for only three sixth of constructs measured. However, some researchers are of the view that a value of 0.5 is adequate to consider as satisfactory correlation [37]. When less stringent criterion of 0.5 is used, the items assessing hyperactivity and impulsivity, abnormalities of communication and restricted, stereotyped behaviour (Cronbach's alpha 0.651-0.691) also can be considered as correlating satisfactorily, confirming the reliability of the instrument.

## Conclusions

The CBAI is a valid and a reliable screening instrument that could be used to identify behavioural problems of 4-6 year age children in the community setting of Sri Lanka. The study supports the preliminary use of the CBAI as a screening instrument and associated with further validation studies undertaken in different community settings.

## Competing interests

The authors declare that they have no competing interests.

## Authors' contributions

**DCS **contributed in conception and design of the study, co-wrote the grant application, responsible for acquisition of data, performed statistical analysis and interpretation of data and prepared the manuscript. **DNF **contributed in the design of the study, interpretation of data and reviewed the manuscript for important intellectual content. **HP **participated in the design of the study, interpretation of the data and reviewed the manuscript for important intellectual content. **RJM **contributed in interpretation of the data and reviewed the manuscript for important intellectual content **HDS **participated in the design and coordination of the study, co-wrote the grant application and reviewed the manuscript for important intellectual content. All authors contributed to the writing of the manuscript and approved the final version.

## Authors' information

**DCS **(MBBS, MSc., MD in Community Medicine) is currently holding the position of post doctoral scholar at the Monash University Accident Research Centre. **DNF **Senior Professor in Community Medicine. **HP **Professor in Psychological Medicine. **RJM **Professor in Epidemiology and the Director of Monash University Accident Research Centre. **HDS **(MBBS, MSc., MD in Community Medicine)

## Supplementary Material

Additional file 1 **Child Behaviour Assessment Instrument**. The CBAI including instructions for interviewers. Click here for file
