# Supplementary material for: The Child Behaviour Assessment Instrument: development and validation of a measure to screen for externalising child behavioural problems in community setting
Source: Int J Ment Health Syst. 2010 Jun 8;4:13. doi: 10.1186/1752-4458-4-13 (PMC2897774; doi:10.1186/1752-4458-4-13)
Supplement: Additional file 1 — Child Behaviour Assessment Instrument. The CBAI including instructions for interviewers. [file 1752-4458-4-13-S1.PDF]

Child Behaviour Assessment Instrument

Now I am going to read some behavioural patterns of the children of age 4-6 years. Please listen carefully and tell me the answers that best describe your child's behaviour over the past six months.

	Behavioural pattern	Very often	Some times	Never
1	Can maintain attention in simple tasks or play activities			
2	Does not complete simple tasks			
3	Struggles (runs about or climbs things) excessively in situations where it is inappropriate			
4	Does not await for his turn in playing or other activities			
5	Follows instructions of adults			
6	Purposely destroys property (toys, books, ornaments)			
7	Hurtful (bites, hits, pinches) to others			
8	Purposely harm (bites, head banging) himself			
9	Unable to control temper (shouts, screams, snap feet when gets angry)			
10	Eye to eye contact is present when talking to others			
11	Plays easily with other children			
12	Shows abnormal body movements (rocking, spinning, strange finger movements)			
13	Gets upset when routine change			
14	Speech is relevant to the occasion			
15	When playing imitates adults			

Please tell me if you have any other behaviours concerned of your child.

Instructions to interviewer

- Identify the parent or the principal caregiver of the child. Ask questions only from the questionnaire by using same wordings and try to avoid your explanations.
- Do not suggest answers to the interviewees.
- Record the answer by marking (√) on the appropriate cage given in front of each question

Scoring system of the CBAI

Item No	***Scoring***		
	**Very often**	**Some times**	**Never**
2,3,4,6,7,8,9,12,13	2	1	0
1,5,10,11,14,15	0	1	2

A total score of $\geq$16 may indicate the presence of an externalising behaviour problem. Please refer the child for professional care.
